# *Omics* technologies in aquafeed: unlocking the black box towards systems biology

**DOI:** 10.1007/s10142-026-01937-6

**Published:** 2026-06-16

**Authors:** Mustafa Öz, Enes Üstüner

**Affiliations:** Department of Fisheries and Diseases, Faculty of Veterinary Medicine, Aksaray University, Aksaray, Türkiye

**Keywords:** Precision aquafeed, Systems biology, Multi-*omics* integration, Nutrigenomics, Gut microbiome, Fish health, Sustainable aquaculture, Alternative protein sources

## Abstract

The aquaculture industry is undergoing a critical transition from marine-based to plant-based and novel protein sources. However, the physiological impacts of these dietary shifts remain largely obscured when evaluated solely by traditional performance metrics such as Feed Conversion Ratio (FCR) and Specific Growth Rate (SGR). This ‘Black Box’ approach fails to detect sub-clinical metabolic disorders, gut dysbiosis, and molecular stress responses until phenotypic losses occur. This review provides a comprehensive synthesis of how omics technologies – nutrigenomics, proteomics, metabolomics, and metagenomics – are elucidating the molecular mechanisms underlying fish nutrition. We examine the capacity of transcriptomics to identify early markers of soybean meal-induced enteritis and the role of proteomics in assessing muscle quality beyond mere gene expression. Furthermore, we highlight the integration of these layers into a ‘Systems Biology’ approach, utilizing multi-omics and bioinformatics to unravel the complex diet-microbiota-host axis. Finally, the review discusses the transition towards ‘Precision Aquafeed.’ It identifies the current challenges in cost, data standardization, and bioinformatics that must be overcome to implement these high-throughput tools in commercial feed formulation.

## Introduction

The aquaculture industry needs to solve a dual problem which requires both increased production for worldwide protein needs and decreased usage of limited marine resources (New 2002). The sustainability requirement has brought a total transformation to aquafeed production because scientists now use plant proteins and insect meals and additional new ingredients instead of traditional fishmeal (Fan et al., 2022; Li et al., 2023). Beyond protein sources, the replacement of fish oil with terrestrial vegetable oils has further compromised the nutritional integrity of farmed fish, driving a progressive ‘nutritional erosion’ characterized by declining EPA and DHA levels in fillets a challenge that microalgal biorefinery systems are increasingly being proposed to resolve (Öz 2026). The use of alternative ingredients in aquaculture production leads to the presence of anti-nutritional factors (ANFs) and unbalanced nutrient profiles which causes health issues for farmed aquatic species. Research shows that soybean meal containing saponins cause damage to intestinal walls and immune system stability in carnivorous animals (He et al., 2020; Zhang et al., 2021a). The industry depends on (SGR) and (FCR) as its primary assessment tools to evaluate new feed materials although these methods have proven to be challenging.

While informative, these economic modeling metrics operate as a ‘Black Box’ by reporting growth results without revealing the underlying biological mechanisms. Such approach faces a major drawback because metabolic disorders which occur below clinical levels together with oxidative stress and gut bacterial imbalance normally appear before they cause any noticeable growth problems. Recent studies show that animals develop normally, yet their bodies initiate pro-inflammatory responses through NF-κB and TNF-α pathways, while nutrient absorption genes become less active (Zhang et al.,^2021^). The genetic profile of fish determines their food response because rainbow trout (*Oncorhynchus mykiss*) isogenic lines demonstrate different capabilities to handle lipid metabolism and preserve energy stability when consuming plant-based diets (Callet et al., 2021). The development of aquaculture requires scientists to move from using empirical feeding methods to understanding the mechanical processes which control fish growth.

The black box can be unlocked through the use of “*Omics*” technologies which include transcriptomics, proteomics, metabolomics, metagenomics, and nutrigenomics. For example, (Seibel et al. 2022a, 2022) performed research which demonstrated that transcriptomics technology identifies blood biomarkers which emerge before nutritional stress causes clinical disease (Seibel et al., 2022; Zheng et al., 2024). Thus, the final phenotype does not always match gene expression levels because cells use post-transcriptional regulation to control gene expression. Both indicate that proteomics is essential for linking genetic data with protein synthesis, as it enables researchers to study, for example, both flesh quality and muscle growth mechanisms, among others (Buccitelli and Selbach 2020) Wang et al., 2022). Such studies can be further supported by metabolomic approaches that analyze lipid subclasses and metabolic pathways to determine their influence on host physiology (Cao et al., 2022; Deng et al., 2025), while metagenomics elucidates the diet-microbiota-host axis, revealing how feed formulations shape the gut ecosystem (Rimoldi et al., 2021).

The aquaculture field lacks a complete Systems Biology framework which unites multiple data layers from *omics* studies. Research studies currently concentrate on individual technologies; yet, they do not demonstrate how the host genome interacts with the gut microbiome and metabolome, a limitation that necessitates the multi-*omics*integration strategies discussed in Sect. 3.1 (Jendoubi 2021) Pan et al., 2025). These analytical platforms currently operate in isolation; this lack of integration creates a ‘Bioinformatics Bottleneck’ characterized by the generation of vast datasets that fail to yield actionable nutritional solutions. The development of “Precision Aquafeed” remains possible because scientists can create individualized fish feed based on genetic characteristics and life stages but no one has put this technology into practice.

The review connects present *omics* technology advancements to their usage for studying fish nutrition. The discussion advances from studying biological information at individual layer levels to present a unified systems biology framework which combines transcriptomics with proteomics and metabolomics and microbiomics. This study establishes a framework for designing precision aquafeeds that leverage advanced technologies to enhance sustainability and animal health, driven by molecular nutrient processing insights and the implementation of standardized data normalization protocols.

## The *omics* toolbox in fish nutrition

### Nutrigenomics & transcriptomics

**Fig. 1:**
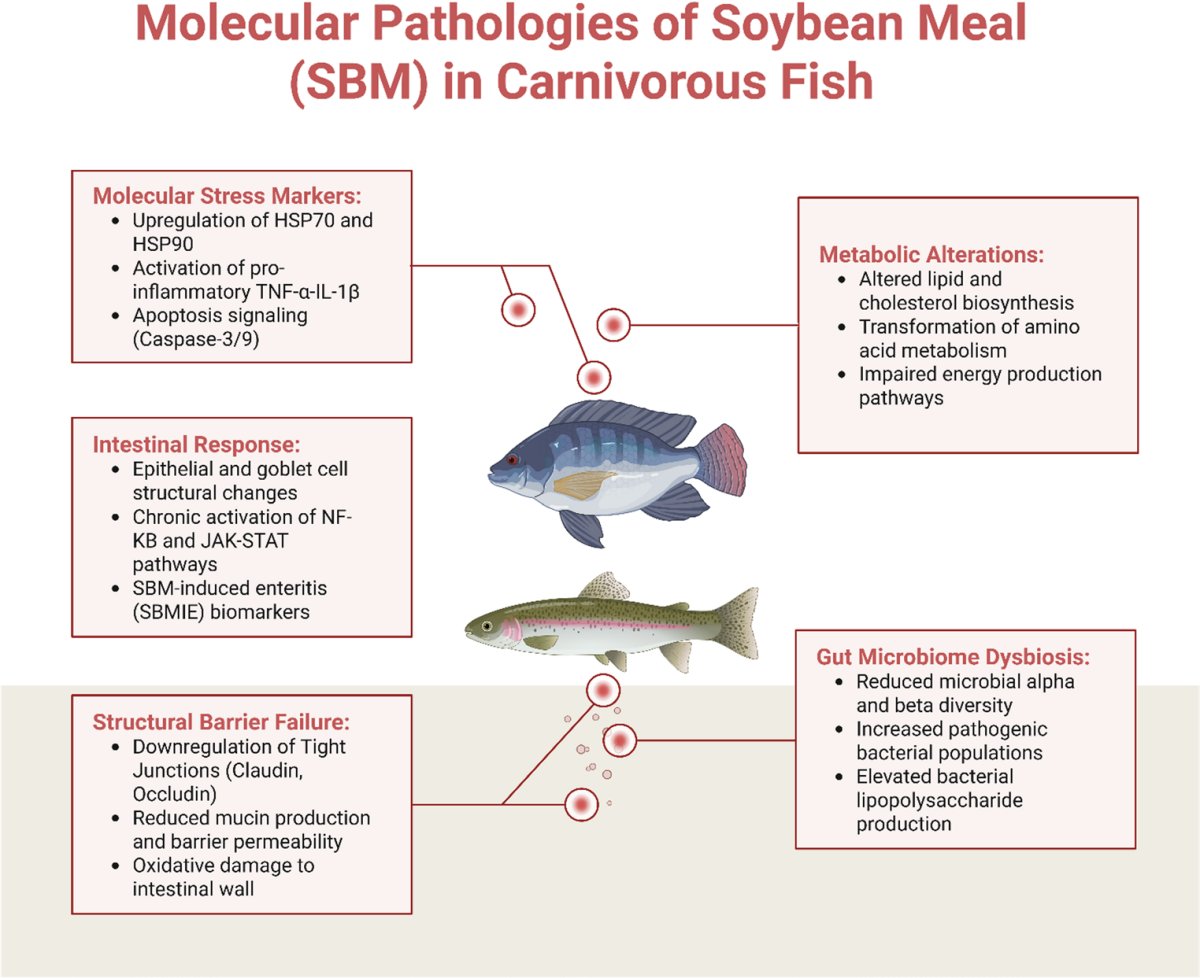
Molecular pathology map of soybean meal-induced disorders in carnivorous fish. The integration of *omics* data reveals that SBM-induced enteritis is not an isolated gut event but a systemic challenge involving molecular stress markers, metabolic shifts in the liver, and significant gut microbiome dysbiosis. These sub-clinical changes often remain hidden in the ‘Black Box’ of traditional growth performance metrics until significant damage occurs

Soybean meal-induced enteritis (SBMIE) in fish is transcriptomically characterized by the strong activation of intestinal innate and adaptive immune pathways. Key signaling includes Toll-like, NOD-like, and RIG-I-like receptors, alongside downstream activation of NF-κB, JAK–STAT, and IRF. This response involves the upregulation of pro-inflammatory cytokines (e.g., *tnf-α*, *il-1β*, *il-6*, *il-8*), chemokines, and interferons. Concurrently, tight-junction components and nutrient digestion/absorption genes are significantly downregulated (He et al. 2020; Zhang et al. 2021a, 2021b, 2021c). This review demonstrates that soybean meal (SBM), soy protein concentrates, and anti-nutritional factors (ANFs) such as saponins, isoflavones, and phytic acid induce significant alterations in epithelial and goblet cell structures. These dietary components suppress mucin production and downregulate key tight junction genes, including*claudins*, *occludin*, and *ZO-1*. Furthermore, ANF-induced oxidative damage leads to cell death, while concurrently modifying cholesterol metabolism, metabolic gene expression, and immune-mediated cytokine production in carnivorous species (He et al. 2020; Zhang et al. 2021a, 2021b, 2021c). These ANF-induced modifications generate biomarkers that extend from gut tissues to the systemic circulation, where they are detectable in blood samples of SBM-fed rainbow trout (*Oncorhynchus mykiss*) and turbot (*Scophthalmus maximus*). For instance, the blood of SBM-fed trout contains immune genes (e.g., *map3k1*, *nod1*, *stat1*, and *hsp90ab*) that show significant potential as non-invasive markers for SBMIE and enteric stress (Seibel et al. 2022a, 2022; Zheng et al. 2024). The genetic profile of fish significantly determines their response to plant-based nutrition. Rainbow trout (*Oncorhynchus mykiss*) isogenic lines capable of surviving on plant-based diets maintain their hepatic capacity for energy and protein production, as well as lipid and cholesterol regulation (Callet et al. 2021). Conversely, sensitive lines exhibit decreased feed conversion efficiency. Moreover, the European sea bass (*Dicentrarchus labrax*) selection program showed that fish maintaining normal distal-intestine inflammatory cytokine production (e.g., *il-1β*, *tnf-α*, and *il-10*) and stable microbiota patterns exist in wild-type populations when consuming ‘future’ diets with low fishmeal content (Torrecillas et al. 2023). Furthermore, the zebrafish (*Danio rerio*) immune-related gene variants (*aif1l* and *arid3c* and *cst14b.2*) function as tolerance markers which enable scientists to forecast improved growth rates in fish that eat SBM+saponin diets (Ulloa et al. 2024). The research data indicates that SBMIE maintains its distinct pattern to some extent because of PRR/NF-κB activation, which disrupts digestive and metabolic functions (He et al. 2020; Zhang et al. 2022, 2022b). Such research findings demonstrate that immune and metabolic system activation patterns between different strains and species maintain their individual characteristics which makes it impossible to find shared indicators for this condition. A comparative overview of these high-throughput technologies, detailing their target molecules, key applications, advantages, and limitations in the context of aquafeed research, is presented in Table 1. The complex molecular pathologies triggered by the dietary shift toward soybean meal, ranging from structural barrier failure to systemic metabolic alterations, are categorized in Fig. 1.

**Table 1:** Comparative overview of *omics* technologies (transcriptomics, proteomics, metabolomics, and metagenomics) applied in aquafeed research, highlighting their target molecules, key applications, advantages, and limitations

Omics Technology	Target Molecules	Key Applications in Aquaculture Nutrition	Advantages	Limitations	Citations
Transcriptomics	mRNA (transcripts)	Identifying gene expression changes due to diet- Biomarker discovery; Feed optimization; Immune response profiling	Reveals active biological pathways- Detects early molecular responses- Useful for non-model species	Does not capture post-transcriptional regulation- Requires high-quality RNA- Data interpretation complexity	(Chandhini and Rejish Kumar 2019; Hakim et al. 2018; Martin and Król 2017; Porter et al. 2022; Zhang 2025)
Proteomics	Proteins, peptides	Assessing protein expression and modifications; Nutritional status assessment; Disease management; Feed efficiency studies	Directly measures functional molecules- Detects post-translational modifications- Links genotype to phenotype	Sample preparation is complex- Lower throughput than transcriptomics- Protein databases may be incomplete	(Carrera 2022; Desouky et al. 2024; Jaiswal et al. 2023; Nandi et al. 2025; Zhang 2025)
Metabolomics	Small metabolites (amino acids, lipids, carbohydrates, organic acids)	Nutritional status monitoring- Feed formulation impact; Biomarker identification; Health and stress assessment	Provides a snapshot of physiological state- Non-invasive sampling possible- Sensitive to subtle changes	Metabolite identification can be challenging- High cost of analytical platforms- Data integration issues	(Alfaro and Young 2018; Dias et al. 2025; Esmaeili et al. 2023; Lulijwa et al. 2022; Roques et al. 2020)
Metagenomics	Microbial DNA (microbiome genes)	Gut microbiome profiling- Understanding host-microbe-nutrition interactions; Probiotic/prebiotic evaluation; Disease resistance	Uncovers microbial community structure and function- Identifies novel genes and pathways- Links diet to microbiome shifts	Requires advanced bioinformatics- Functional annotation is limited- High sequencing costs	(Limborg et al. 2018; Sundaray et al. 2022; Wu et al. 2025; Yukgehnaish et al. 2020; Zhang 2025)

### Proteomics

Fish diet-omics research demonstrates that mRNA levels do not directly correlate with protein abundance because post-transcriptional mechanisms such as translation efficiency, protein degradation, and modification processes prevent transcript levels from effectively predicting changes in functional proteins (Buccitelli and Selbach 2020; Cope et al. 2025). The current understanding of protein changes in functional proteins remains insufficient because transcript levels do not effectively predict protein modifications (Buccitelli and Selbach 2020; Cope et al. 2025). In crucian carp (*Carassius carassius*) and grass carp (*Ctenopharyngodon idellus*), both the muscle proteome and flesh quality are heavily influenced by the concentration and origin of dietary proteins. These traits reach an optimal state when protein levels activate myogenic regulatory factors including *myod*, *myf5*, *myog*, and *mrf4* while concurrently stimulating PI3K–Akt–Smad signaling and antioxidant enzyme production at both the mRNA and protein levels. Consequently, balanced protein levels enhance texture by increasing hardness and collagen production. Conversely, protein levels outside the optimal range impair nuclear factor erythroid 2-related factor 2 (*nrf2*) signaling and autophagy markers such as forkhead box O1 (*foxo1*) and microtubule-associated protein 1 light chain 3 (*lc3*), leading to poor texture and reduced oxidative stability (He et al. 2020; Wang et al. 2022). Thus, substitution of fishmeal with different plant-based and innovative protein sources (soy, rapeseed, cottonseed, chlorella, and insect meals) causes changes in the muscle protein biosynthesis pathways, intramuscular fat, collagen metabolism, and flavor-related amino acids, which results in better muscle characteristics and antioxidant properties but leads to decreased growth rates and reduced protein content because of amino acid imbalances (Fan et al. 2022; Li et al. 2023; Yang et al. 2024). For example, ın low fishmeal/fish-oil formulations, proteomic and related molecular markers of stress and immune challenge include several key categories. These encompass heat shock proteins (*hsp70*/*hsp90*), antioxidant enzymes (e.g., SOD, CAT, GPx), and apoptosis regulators such as caspase-3. Additionally, ER-stress proteins and innate-immune mediators including *tlr*, *tnf*, *il-1β*, and IgM serve as critical indicators whose expression patterns are modulated by lipid levels, functional additives, or plant/insect replacements (He et al. 2023; Huang et al. 2022; Kumar et al. 2024; Serradell et al. 2020; Yu et al. 2023). In summary, these findings highlight that protein, transcript, and phenotype data must be analyzed collectively, as mRNA–protein relationships often emerge only under specific biological conditions. It is also noteworthy that positive shifts in the muscle proteome resulting from alternative protein sources can sometimes lead to negative impacts on overall animal growth and health stability. Furthermore, while proposed proteomic stress biomarkers from low-fishmeal (low-FM) diets show potential, their efficacy is strictly contingent upon specific diet, species, and stressor conditions, necessitating extensive cross-species testing to achieve universal adoption in the aquaculture industry.

### Metabolomics

Metabolomics can uncover the intricacies of fish lipid metabolism by monitoring how dietary lipid subclasses transform into derivative compounds, which subsequently disseminate across various tissues and the systemic circulation (Deng 2025; Gao et al. 2025; He et al. 2025). Research shows that dietary n-3 highly unsaturated fatty acids (HUFA), bile acids, and lysophospholipids influence not only the distribution of triacylglycerols (TAG) and phospholipids, but also the β-oxidation, cholesterol, and energy metabolism pathways in fish liver and muscle tissues (Deng 2025; Gao et al. 2025; He et al. 2025). For instance, the metabolic profiles of rainbow trout (*Oncorhynchus mykiss*) and turbot (*Scophthalmus maximus*) reveal dietary differences between marine and plant/terrestrial sources through Proton Nuclear Magnetic Resonance (^1^H-NMR) and Mass Spectrometry (MS)-based metabolomics. These analyses identify significant shifts in amino acids, choline, taurine, purine intermediates, and energy-related metabolites. Such metabolic signatures demonstrate that fish that consume plant-based diets undergo a transformation in their amino acid metabolism, leading to a lipid metabolism profile that is distinct from those fed traditional marine ingredients (Cao et al. 2022; Deborde et al. 2021; Hoerterer et al. 2023). Furthermore, plasma metabolomics together with transcriptomics or proteomics analysis provides researchers with a new non-invasive method for research. The NMR-based plasma profiling method enables researchers to detect dietary imbalances and plant-induced enteritis in rainbow trout (*Oncorhynchus mykiss*) (Deborde et al. 2021; Palma et al. 2021). Moreover, the analysis of Atlantic salmon (*Salmo salar*) and meagre (*Argyrosomus regius*) plasma through metabolomics reveals that lipid species and various metabolites function as indicators which help scientists understand infection status and nutritional health and complete biological condition (Hoel et al. 2025; Ivanova et al. 2024; Oliveira et al. 2025). The metabolic response of fish to plant-based diets with low fishmeal and oil content leads to distinct cholesterol regulation patterns. Furthermore, these diets result in decreased lipid absorption and storage alongside elevated triglyceride release, with metabolic adjustments for amino acids and energy varying according to fish species and dietary composition (Angelakopoulos 2025; Angelakopoulos et al. 2025b; Cao et al. 2022; Villasante et al. 2025). Metabolomics data provides critical insights into how**fish** metabolize marine-derived nutrients relative to plant-based alternatives. The development of plasma (or mucus) metabolite panels for aquaculture lipid nutrition diagnosis needs additional validation through research with different species while requiring performance and histology data integration and established sampling protocols.

### Metagenomics & microbiomics

**Fig. 2:**
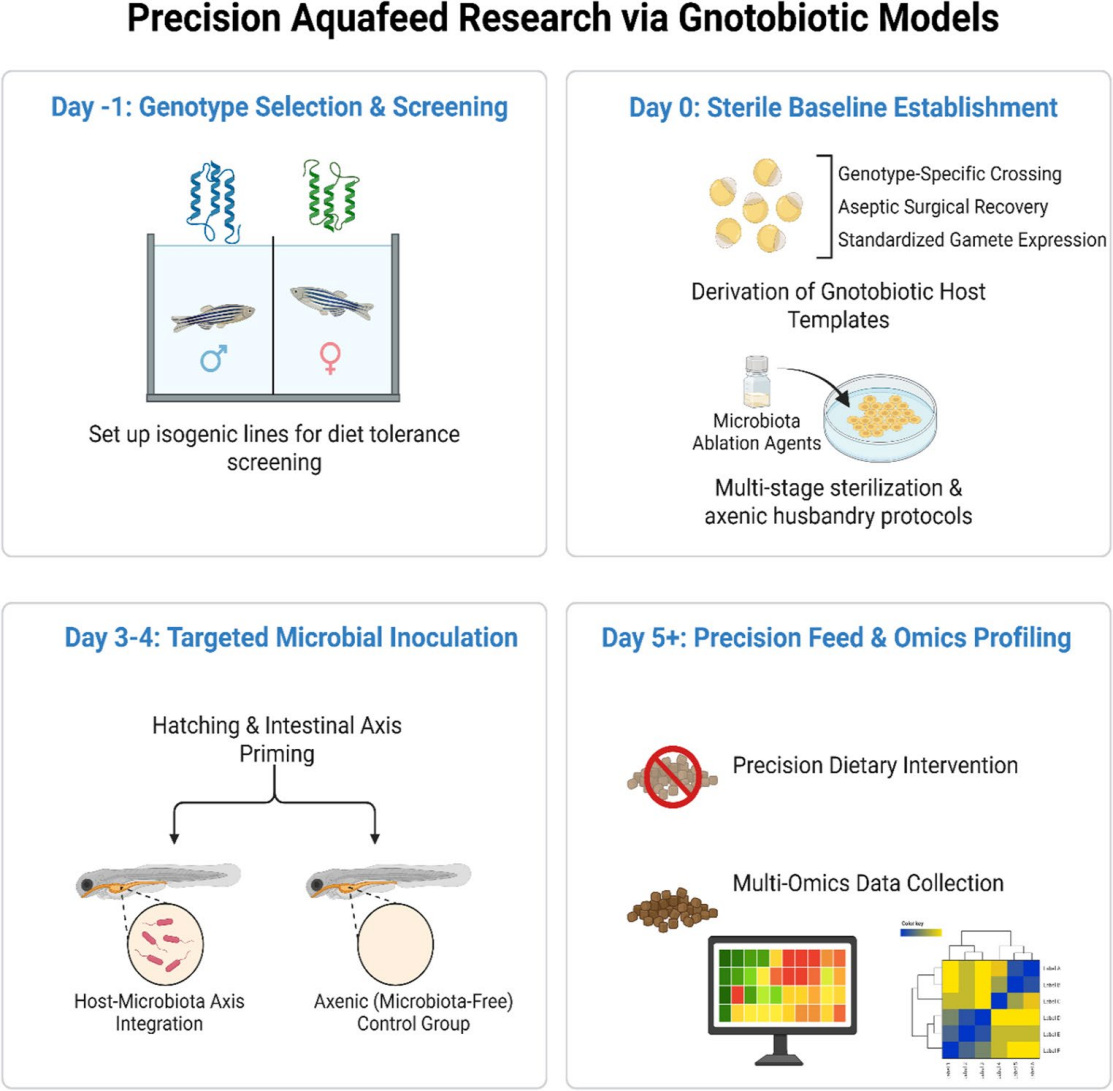
Methodological framework for unlocking the nutritional “Black Box” using gnotobiotic Zebrafish models. (Day − 1): Selection of specific isogenic lines to evaluate dietary tolerance or sensitivity across different genotypes. (Day 0): Establishing a sterile molecular baseline through aseptic surgical recovery or standardized gamete expression to isolate host molecular responses from microbial interference. (Day 3–4): Targeted inoculation of specific bacterial communities to investigate diet-microbiota-host axis interactions and priming of the intestinal transcriptome. (Day 5+): Integration of Precision Aquafeed formulations followed by high-throughput multi-*omics* profiling (transcriptomics, proteomics, and metabolomics) to identify sub-clinical biomarkers and metabolic shifts

When aquaculture species switch from fishmeal to plant, insect, or poultry by-product proteins and functional additives, the composition of feed ingredients determines the resulting gut microbiota diversity (Rimoldi et al. 2021, 2020; Serra et al. 2021). This substitution leads to permanent changes in alpha and beta diversity, as well as the distribution of*Firmicutes*, *Proteobacteria*, *Fusobacteria*, and *Bacteroidetes*. Digesta-associated microbial communities exhibit higher sensitivity to dietary shifts than the core microbial community, which resides in the mucosa (Rimoldi et al. 2021, 2020; Serra et al. 2021). These shifts have functional importance because feeding habits and dietary composition foster communities of carbohydrolytic, proteolytic, and lipid-degrading microorganisms. These changes affect digestive enzyme performance and energy, amino-acid, and lipid metabolism, ultimately resulting in improved growth rates and feed efficiency (Bera et al. 2023; Rai et al. 2025). The use of antibiotics to disrupt gut microbiota results in decreased growth rates and impaired protein and lipid metabolic processes. Conversely, a stable microbiota community improves nutrient processing and cell growth signals, leading to better growth outcomes (Kong et al. 2024). High plant protein diets and plant-derived dietary regimes often lead to dysbiosis, which is characterized by decreased microbial diversity and increased pathogenic bacteria. These conditions elevate bacterial lipopolysaccharide production, which causes enteritis and impairs gut function (Bera et al. 2023; Serra et al. 2021). Prebiotics including galactomannans, agavin, Aloe-polysaccharides, and inulin help restore imbalanced gut microbiota by enhancing populations of*Lactobacillales* and *Bacillales*, as well as butyrate-producing bacteria. This restoration occurs while decreasing *Vibrionales*/coliforms and enhancing gut structure and metabolic pathways, resulting in improved health outcomes (Bera et al. 2023; De Marco et al. 2023). Research confirms the intricate relationships between diet, the microbiome, and the host. However, utilizing prebiotic therapy to mitigate plant-based diet-induced microbiome disturbances requires precise, species-specific dosages to effectively enhance feed efficiency. A summary of key transcriptomic biomarkers associated with plant-based diet induced enteritis is provided in Table 2. To establish cause-and-effect relationships within the diet-microbiota-host axis, researchers utilize gnotobiotic models like Zebrafish, allowing for the precise molecular monitoring of host responses to specific feed components, as detailed in Fig. 2.

**Table 2:** Key transcriptomic biomarkers and gene expression alterations associated with soybean meal-induced enteritis (SBMIE) and plant-based diets in carnivorous fish species

Fish Species/Model	Key Biomarkers/Genes (Up/Down)	Expression Pattern in Enteritis/Inflammation	Associated Pathways/Notes	Citations
Atlantic salmon (*Salmo salar*)	*IL-1β*,* IL-8*,* TNF-α*,* GTPase IMAP*,* NF-κB*,* S100V2*,* S100I2*	Upregulated pro-inflammatory genes (*IL-1β*,* IL-8*,* TNF-α*,* S100V2*); *S100I2* variable	Immune response, T/B cell regulation, lipid metabolism, ion transport, taurine/hypotaurine metabolism, cytochrome *P450*, steroid biosynthesis	(Kiron et al. 2020; Kortner et al. 2012; Król et al. 2016; Sahlmann et al. 2013; Tsai et al. 2023)
Rainbow trout (*Oncorhynchus mykiss*)	*MAP3K1*,* LYG*,* NOD1*,* STAT1*,* HSP90AB*,* S100I2*,* S100V2*	Upregulation of stress/inflammatory markers (*MAP3K1*,* NOD1*,* S100V2*); *S100I2* variable	Inflammatory response, calcium homeostasis, immune signaling	(Blaufuss et al. 2019; Seibel et al. 2022a, 2022)
Turbot (*Scophthalmus maximus*)	*tnf-α*,* il-6*,* il-8*,* tgf-β*,* il-10*,* claudin3/4/7*,* jak1/2b*,* stat1/5a*,* tlr2/5b*	Upregulation of pro-inflammatory cytokines (*tnf-α*,* il-6*,* il-8*,* jak/stat*,* tlr*); anti-inflammatory cytokines (*tgf-β*,* il-10*) up with mitigation	*JAK-STAT*, *TLR*, tight junction, *IBD* pathways	(Tan et al. 2019; Yu et al. 2021; Zheng et al. 2024)
Pearl gentian grouper (*Epinephelus fuscoguttatus* × *E. lanceolatus*)	*IL-1β*,* IL-8*,* IL-17*,* TNF-α*,* CSF1*,* IL-4*,* IL-10*,* TGF-β1*,* TLRs*,* NODs*,* RIGs*	Upregulation of pro-inflammatory (*IL-1β*,* IL-8*,* IL-17*,* TNF-α*,* CSF1*); Downregulation of anti-inflammatory (*IL-4*,* IL-10*,* TGF-β1); TLR/NOD/RIG* activation	*TLR-MyD88-NF-κB*,* PRR* signaling, immune network for IgA, tight junction disruption	(He et al. 2020; Pang et al. 2023a, 2023b)^2024^; Zhang et al. 2021a, b, c, 2022a, b, 2023
Largemouth bass (*Micropterus salmoides*)	*TNF-α*,* IL-1β*,* ACC*,* caspase-3/8/9*,* TOR*,* eIF4E-BP*,* ZO-1*,* occludin*	Upregulation of pro-inflammatory (*TNF-α*,* IL-1β*,* caspases*); Downregulation of tight junction proteins (*ZO-1*, *occludin*)	Inflammation, apoptosis, tight junction, protein synthesis	(Chen et al. 2024; (Weng et al. 2023; Zhao et al. 2023)
Olive flounder (*Paralichthys olivaceus*)	*ERS* genes (e.g., *GRP78*, *CHOP*), *TNF-α*,* IL-1β*,* IL-10*,* TGF-β1*,* claudins*,* occludin*	Upregulation of *ERS* and pro-inflammatory genes; Downregulation of anti-inflammatory and tight junction genes	*ER* stress, inflammation, apoptosis, barrier function	(Su et al. 2025)
Japanese seabass (*Lateolabrax japonicus*)	*TNF-α*,* IL-1β*,* IL-2*,* IL-8*,* IL-4*,* PepT1*,* LAT1*,* SLC1A5*	Upregulation of pro-inflammatory (*TNF-α*,* IL-1β*,* IL-2*,* IL-8*); Downregulation of anti-inflammatory (*IL-4*)	Inflammation, nutrient transport	(Zhang et al. 2018)
Zebrafish (*Danio rerio*)	Downregulation: immune genes (e.g., *il1b*,* tnfa*,* cxcl8a*,* mmp9*,* mmp13a*,* mmp14b*,* mmp17b*,* mmp28a*); Upregulation: retinol signaling, *SREBP*, cholesterol catabolism	Tolerant strains: dampened immune gene expression, upregulated lipid metabolism	Immune suppression, lipid metabolism, retinol signaling	(Valenzuela et al. 2021)
Common carp (*Cyprinus carpio*)	Inflammation-related genes, antioxidant genes, tight junction proteins	Upregulation of pro-inflammatory, downregulation of anti-inflammatory and barrier genes	Inflammation, oxidative stress, barrier function	(Xie et al. 2021, 2021b)

*ACC* acetyl-CoA carboxylase, *CSF1* colony stimulating factor 1, *ER* endoplasmic reticulum, *ERS* endoplasmic reticulum stress, *IBD* Inflammatory bowel disease, *IL* interleukin, *JAK-STAT* janus kinase-signal transducer and activator of transcription, *MAP3K1* mitogen-activated protein kinase kinase kinase 1, *NOD* nucleotide-binding oligomerization domain, *PRR* pattern recognition receptor, *RIG* retinoic acid-inducible gene, *SREBP* sterol regulatory element-binding protein, *TGF-β* transforming growth factor beta, *TLR* toll-like receptor, *TNF-α* tumor necrosis factor alpha, *ZO-1* zonula occludens-1

## Systems biology: the holistic approach

### Multi-*omics* integration

The combination of transcriptomics with proteomics and metabolomics in fish nutrition creates a unified view that reveals how dietary components affect gene expression, protein networks, and metabolic outcomes beyond the reach of individual analyses. This integrated system demonstrates how dietary components trigger sequential changes in energy, lipid, and amino acid metabolism, as well as stress response pathways, which cannot be observed through separate analysis of each component (Du et al. 2023; Jendoubi 2021; Natnan et al. 2021; Pan et al. 2025; Sanches et al. 2024). Nutrition-focused aquaculture research combines transcriptome–metabolome and proteome–metabolome analyses to evaluate fishmeal-free diets and alternative protein sources. These studies demonstrate how such diets affect feed efficiency through specific genes, enzymes, and metabolic pathways that control digestive performance, lipid utilization, and growth rate in plant-based feed systems (Cao et al. 2022; Jendoubi 2021; Pan et al. 2025). For example, the host–microbiome axis is explored through multi-omics analysis using metagenomics/metatranscriptomics and untargeted metabolomics. In rainbow trout (*Oncorhynchus mykiss*), this approach reveals how probiotic/synbiotic feeds transform gut microbial populations while shifting microbial metabolic pathways. These changes alter intestinal lipid, bile acid, and amino-acid metabolite profiles, which subsequently affect host metabolic processes (Rasmussen et al. 2022). Current research directly connects gut microbiome information with host gene expression patterns via integrative multi-omics frameworks. In grass carp (*Ctenopharyngodon idellus*), metagenomic patterns involving *Proteobacteria* versus *Fusobacteria*, *Firmicutes*, and *Bacteroidetes*are linked to intestinal and hepatic transcriptional modules and short-chain fatty acid (SCFA)-sensitive host genes (Li et al. 2024). Recent evidence from fish and crustaceans demonstrates that intestinal microbial communities show statistical relationships with immune and metabolic genes, which determine disease resistance and growth patterns (Zhao et al. 2025; Zheng et al. 2025). While the combination of multiple omics techniques enables a better understanding of dietary responses, establishing cause-and-effect relationships remains a challenge. To bridge the gap between observed correlations and causative mechanisms, future research must prioritize experimental approaches such as gnotobiotic models and targeted perturbation experiments. Furthermore, standardized experimental designs and predictive modeling are essential to develop effective precision-nutrition tools for aquaculture (Fajardo et al. 2022).

### From data to knowledge: bioinformatics

**Fig. 3:**
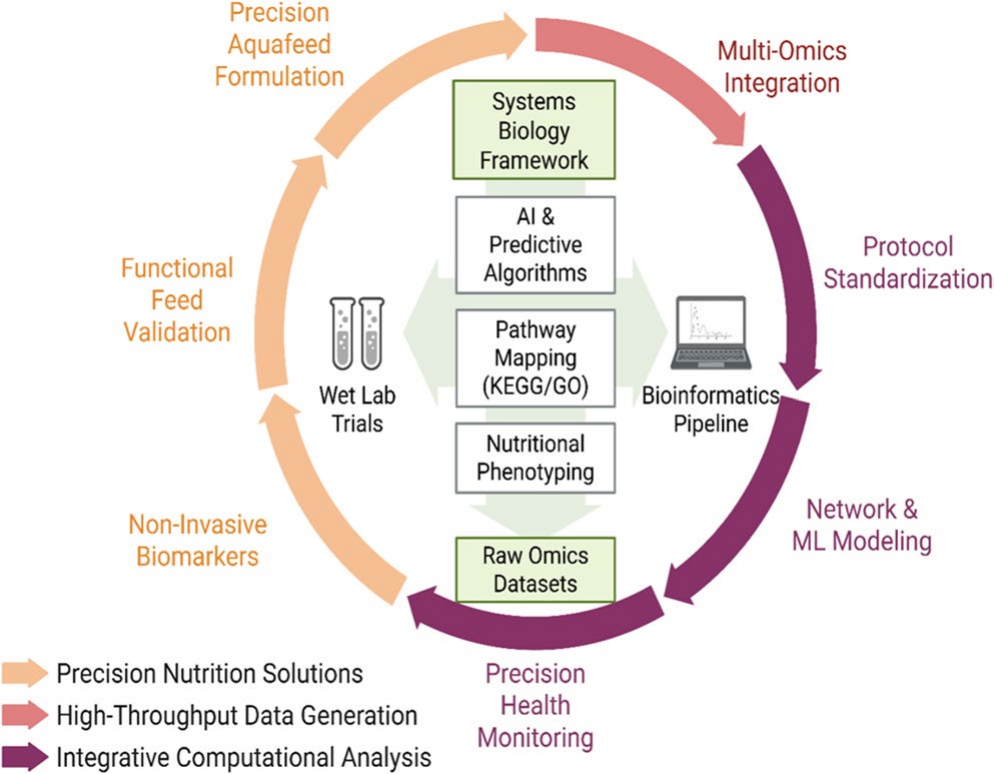
The bioinformatics and data-to-knowledge cycle for precision aquafeed. The outer ring demonstrates the high-throughput process from multi-*omics* integration to the commercial implementation of precision formulations. The central core represents the computational framework required to bridge the bioinformatics bottleneck, utilizing AI algorithms and pathway mapping to transform raw datasets into a holistic systems biology framework. The cycle illustrates the transformation of high-throughput data generation into precision nutrition solutions through integrative computational analysis

To convert omics data into meaningful biological insights, researchers utilize enrichment tools such as the Kyoto Encyclopedia of Genes and Genomes (KEGG), Gene Ontology (GO)-based analysis, and gene set enrichment analysis (GSEA) (Chicco and Agapito 2022; Zhao and Rhee 2023). Network analysis enables the development of interaction graphs and co-expression/co-abundance networks that arrange identified molecules into structured models. This approach organizes nodes (genes, proteins, metabolites) and edges into modules and hubs, revealing critical regulatory systems that standard single-layer analyses fail to identify (Agamah et al. 2022; Ryan et al. 2025). Complex network analysis in aquaculture now encompasses disease spread, ecological interactions, and gene expression patterns. These models allow researchers to identify core network constituents and critical vulnerabilities within production frameworks (Rather et al. 2023; Vidza et al. 2025). Machine learning (ML) systems utilize multi-omics data to discover complex relationships between molecular data and phenotypes, which direct optimization processes. For instance, a two-stage Bayesian optimization framework has been successfully applied to optimize leopard coral grouper (*Plectropomus leopardus*) feed formulations for growth and sustainability (Terayama 2025). The development of Machine Learning (ML)-based multi-*omics*integration and network methods for complex trait prediction and molecular driver identification in other fields demonstrates potential methods which can be applied to aquafeed design (Öz et al. 2025a, 2025b). Critically, while these pathway, network and ML tools greatly enhance interpretation and predictive power, their reliability in fish nutrition depends on input data quality, appropriate background/pathway databases, biological interpretability of networks, and rigorous benchmarking of ML models, without which statistically significant results may fail to yield robust, actionable improvements in aquafeed formulation (Öz et al. 2025a, 2025b). The iterative process of converting high-dimensional*omics* datasets into actionable nutritional knowledge, through the integration of computational modeling and standardized protocols, is systematically presented in Fig. 3. Furthermore, transcriptomic tools enable the detection of nutritional and environmental stress indicators in fish through blood cell transcriptomics, which show promise but require further validation to become standard biomarkers.

## Application: towards precision aquafeed

### Biomarker discovery

The transcriptomic study of red blood cells from gilthead seabream (*Sparus aurata*) that consumed plant-based or fish-based diets revealed that their cells developed identical changes in their oxidative phosphorylation, ribosomal function, and lipid metabolism. These pathways adapted to dietary stress throughout several weeks while following the fish’s extended growth patterns, thereby confirming their function as feed stress markers that appear before disease onset (Angelakopoulos 2025; Angelakopoulos et al. 2025b). Validated metabolomic and proteomic biomarkers for flesh quality and health status are summarized in Table 3. For instance, the multigene panel analysis of rainbow trout blood during soybean meal enteritis identified immune and stress genes (*MAP3K1*, *NOD1*, *STAT1*, *HSP90AB*) which researchers believe could serve as indicators for poor nutrition and gastrointestinal inflammation (Seibel et al. 2022a, 2022). The “Fit-Chip” system for salmon (*Salmo salar*) uses compact gene sets to successfully identify food restriction patterns (Frommel et al. 2025). Research utilizing untargeted and targeted metabolomics to study flesh quality has shown that nucleotide breakdown products are strong indicators of freshness. ATP catabolites including hypoxanthine, inosine, and uracil provide more accurate storage duration metrics than traditional Total Volatile Basic Nitrogen (TVB-N) and Trimethylamine/Trimethylamine Oxide (TMA/TMAO) measurements (Du et al. 2021; Marhuenda-Egea and Sanchez-Jerez 2025). Current efforts focus on the development of needle-free biomarkers utilizing epidermal mucus as a diagnostic medium. Specific miRNAs identified in both plasma and mucus, such as*miR-210-3p*, demonstrate high sensitivity to hypoxia and nutritional stress (Cardona et al. 2022). Furthermore, skin mucus protein analysis, in conjunction with glucose, lactate, and cortisol measurements, provides a non-invasive approach to monitor experimental parameters and ensure animal welfare (Alzate-Díaz et al. 2025; Tejero et al. 2025). Dietary phytogenic oil supplementation, including black cumin and garlic oils, has been consistently demonstrated to mitigate pesticide- and contaminant-induced hematological, biochemical, and histopathological damage in Nile tilapia (*Oreochromis niloticus*), further underscoring the practical value of multi-biomarker frameworks in aquaculture health monitoring (Öz 2024; Öz et al. 2024b) Öz, Üstüner, Jumayeva, et al., 2025b). Additionally, distinct metabolomic patterns within the mucus serve as reliable indicators for evaluating the overall health status of aquatic organisms (Ekman et al. 2023; Ivanova et al. 2021). The existing stress, quality, and gut-related welfare transcriptomic and metabolomic signatures show promise but their application between farms remains limited because they were developed for specific species under particular conditions and their results are mostly based on correlations and lack standardization. Consequently, there is a critical need for large-scale, multi-species validation studies to establish robust diagnostic protocols for the industry. Ultimately, nutrigenomics enables life-stage diet planning by linking dietary interventions to changes in gene expression and metabolic processes during development.

**Table 3:** Validated metabolomic and proteomic biomarkers for assessing flesh quality, freshness, and physiological health status in aquaculture species towards precision nutrition

Biomarker Type	Example Biomarkers/Pathways	Application (Quality, Freshness, Health)	Sample Matrix	Species/Context	Citations
Metabolomic	Creatinine, His, Lys, Ala, Phe, pantothenic acid, phosphoric acid, pyruvate, N-acetylglutamic acid, (lyso)phosphatidylcholines, ceramides, triglycerides	Flesh quality (taste, texture), freshness, disease progression, nutritional status	Muscle, plasma, skin mucus, feces	Salmon (*Salmo salar*), sea bream (*Sparus aurata*), tilapia (*Oreochromis niloticus*), ayu (*Plecoglossus altivelis*), rainbow trout (*Oncorhynchus mykiss*), Chinook salmon (*Oncorhynchus tshawytscha*), and puffer fish (*Takifugu rubripes*)	(Benedetto et al. 2024; Cao et al. 2022; Ivanova et al. 2024; Lulijwa et al. 2022; Men et al. 2020; Roques et al. 2020; Sidira et al. 2024; Takeuchi et al. 2024; Wiens et al. 2023; Young et al. 2023)
Proteomic	*Hsp70*,* Grp78*,* Erp57*,* Gfpt1*,* Pyg*,* Acc1*,* FN1B*,* EIF3C*,* C1q-LP3*, troponin, myosin, complement factor H-like 1, prothymosin alpha, NADH: ubiquinone oxidoreductase, Mx species, β-actin, immune-related proteins	Stress response, health status, malnutrition, disease resistance, post-mortem freshness, muscle growth, food safety	Liver, muscle, plasma, skin mucus, blood, gills, digestive gland	Gilthead seabream (*Sparus aurata*), largemouth bass (*Micropterus salmoides*), meagre (*Argyrosomus regius*), puffer fish (*Takifugu rubripes*), Chinook salmon (*Oncorhynchus tshawytscha*), rainbow trout (*Oncorhynchus mykiss*), and bivalves (Bivalvia)	(de Magalhães et al. 2023; Esmaeili et al. 2021, 2022, 2023; Hematyar et al. 2025; Jia et al. 2022; Men et al. 2020; Oliveira et al. 2025, 2024; Perez et al. 2025) Raposo de Magalhães et al., 2020; (Riva 2025; Wiens et al. 2023)
Enzymatic (Metabolic)	ALP (Alkaline phosphatase), ALT (Alanine aminotransferase), AST (Aspartate aminotransferase), GDH (Glutamate dehydrogenase), ME (Malic enzyme), HOAD (β-hydroxyacyl-CoA dehydrogenase), protease, superoxide dismutase, lysozyme	Health status, stress, nutritional assessment, oxidative stress	Blood, plasma, skin mucus	Multiple finfish and shellfish species	(Bacchetta et al. 2024; Oliveira et al. 2025, 2024; Roques et al. 2020)
Integrated *Omics*	Multi-*omics* panels (proteins + metabolites + lipids), pathway signatures (protein synthesis, degradation, lipid metabolism, immune response)	Feed efficiency, stress adaptation, disease diagnosis, environmental monitoring, food authentication	Liver, muscle, plasma, skin, gills, feces	Chinook salmon (*Oncorhynchus tshawytscha*), sea bass (*Dicentrarchus labrax*), flounder (*Paralichthys olivaceus*), and bivalves (Bivalvia)	(Benedetto et al. 2024; Esmaeili et al. 2021, 2022, 2023; Laurent et al. 2023) Raposo de Magalhães et al., 2022; (Young et al. 2023)
Non-lethal Biomarkers	Plasma proteins, skin mucus proteins, fecal metabolites (cortisol, glucose, acetate), blood parameters (total protein, albumin, triglycerides)	Health and nutritional status, disease detection, stress	Plasma, skin mucus, feces, blood	Meagre (*Argyrosomus regius*), ayu (*Plecoglossus altivelis*), and pacu (*Piaractus mesopotamicus*)	(Bacchetta et al. 2024; Oliveira et al. 2025; Takeuchi et al. 2024)

### Precision formulation

**Fig. 4:**
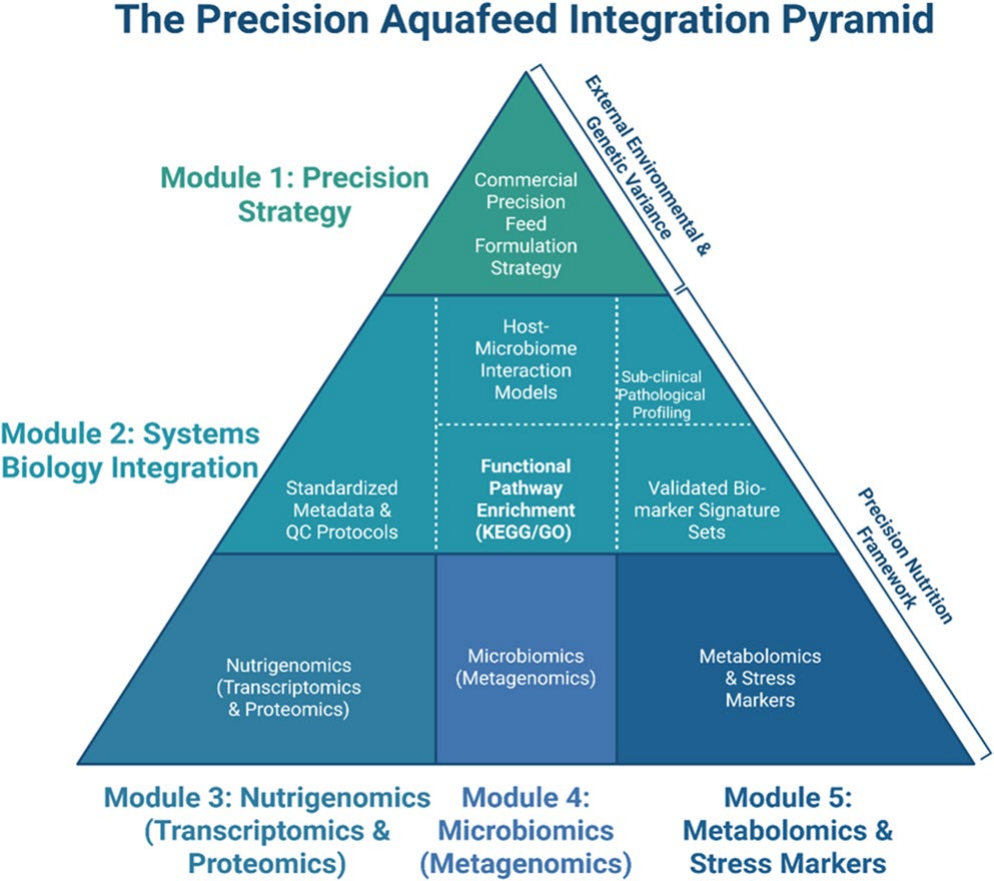
The hierarchical integration pyramid for Precision Aquafeed. This framework illustrates the multi-stage progression from raw data to industrial implementation. Modules 3–5 represent the foundational high-throughput *omics* layers that unlock the molecular ‘Black Box’ of fish physiology. Module 2 acts as the analytical hub where standardized metadata and bioinformatics pipelines synthesize raw data into functional pathways and predictive models. The apex, Module 1, represents the ultimate industrial goal: the delivery of Precision Aquafeed Formulations tailored to specific life stages and genotypes, within a holistic Precision Nutrition Framework that accounts for external environmental and genetic variance

The field of nutrigenomics enables life-stage diet planning through its ability to connect dietary interventions with the way genes express themselves and metabolic processes change during development. Research indicates that these stages require specific protein-energy ratios, digestive enzyme support, and functional additives that are critical at particular life stages (Rønnestad et al. 2013). A study of pike silverside (*Chirostoma estor*) larvae that consumed live prey showed upregulation of growth and digestive function genes. Conversely, microdiet consumption triggered stress and DNA damage responses, leading to the development of improved early-life formulations (Desouky et al. 2024; Juárez-Gutiérrez et al. 2021). The development of aquafeeds tailored to specific genotypes is now feasible.*Omics*technologies allow scientists to identify genetic variations influencing fish growth, disease resistance, lipid metabolism, and gut microbiome interactions. The intestinal tissues of European sea bass and gilthead seabream show different gene expression patterns, immune system reactions, and gut bacterial communities when they receive diet that contains reduced fish meal content. The research shows that particular fish genotypes achieve better results from sustainable feed formulations so scientists need to develop customized functional ingredients and fatty acid compositions for these genotypes (Naya-Català et al. 2022; Rimoldi et al. 2023).*Omics* technologies validate insect meals serve as fish-meal substitutes through multiple assessment methods which include digestibility tests, fatty-acid identification, and molecular health indicator evaluation: *Tenebrio molitor* and *Hermetia illucens*meals show high apparent protein digestibility and complete essential amino-acid availability when used as feed for sea bass and trout (Basto et al. 2020; Carvalho et al. 2023; Mastoraki et al. 2020). The gene expression patterns in transcriptomic and gut health assessments (*hsp70/90*, cytokines, *MHCII*) show stable results or only small changes when participants consume standard amounts of the listed foods (Carvalho et al. 2023). The multi-*omics*and Fatty Acids (FA)-profiling results show that fish tissues develop permanent changes which lead to higher levels of saturated and n-6 fatty acids but lower n-3 LC-PUFA levels; thus, needing either substrate changes or marine oil supplementation to preserve fillet nutritional value (Carvalho et al. 2023; Melenchón et al. 2022; Truzzi et al. 2023). The design of diets benefits from nutrigenomics and*omics* technologies but most research findings apply to specific species under particular conditions without sufficient large-scale testing, complete breeding program integration, insufficient product quality, and health benefit assessments. The development of commercial feeds which match life stages and genotypes of aquaculture species through insect-based formulations remains an essential but not fully achieved goal. The systematic transition from raw molecular data to industrial implementation, incorporating both host-specific and environmental variables, is conceptualized in Fig. 4. This hierarchical integration pyramid outlines the progression from raw data (Module 1) to functional validation (Module 2), leading to precision formulations across all development stages (Modules 3–5).

## Challenges and limitations

### Cost and complexity

The aquafeed industry faces significant obstacles in adopting omics technologies due to high expenses for instrumentation, data analysis, and specialized expertise. These costs create substantial challenges for small-scale operations with limited profit margins. While the technology is applied in research and high-value breeding programs, it remains difficult to implement in standard production (Andersen et al. 2025; Zhang 2025).

Metabolomics provides researchers with more accessible methods to conduct nutritional assessments. For example, NMR-based workflows enable fast, semi-automated plasma profiling for both industrial quality control and routine nutritional monitoring. However, these methods may sacrifice some precision for faster results at reduced per-sample costs once the system is operational (Tavares et al. 2022). Recent studies demonstrate how portable NMR equipment for benchtop use, combined with simplified MS testing methods, can reduce the barriers preventing aquaculture industries from adopting these technologies (Lulijwa et al. 2022). The utilization of non-invasive matrices, such as mucus and urine, enables researchers to conduct extensive field studies cost-effectively while significantly reducing lethal sampling and animal mortality (Ekman et al. 2023). Similarly, multi-biomarker approaches integrating hematological, biochemical, oxidative stress, and histopathological endpoints have proven effective in detecting sub-clinical stress responses in fish exposed to waterborne contaminants, while also highlighting the hepatoprotective potential of dietary phytogenic additives (Öz et al. 2026). Moreover, The environmental metabolomics study of bivalves shows that scientists can conduct site comparisons by using basic experimental methods (Fuller et al. 2024; Hani et al. 2021). Furthermore, high-throughput sequencing methods, including eDNA and diet metabarcoding, enable affordable surveys that produce more information than conventional morphological methods (Evans et al. 2021; Rodríguez-Rodríguez et al. 2022). Aquaculture research still necessitates complete cost-benefit evaluations and conventional nutritional assessments. While genomic approaches are increasingly effective for biodiversity monitoring, they have yet to fully supplant conventional production evaluation methods, such as growth trials and digestibility assays (Lulijwa et al. 2022; Rodríguez-Rodríguez et al. 2022). Ultimately, omics technology achieves its highest economic value through strategic implementation using specific tests to solve critical questions rather than attempting to replace all conventional assessment methods (Andersen et al. 2025; Lulijwa et al. 2022).

### Data standardization

The process of standardizing fish transcriptomic sampling faces multiple obstacles. Different species and tissues show varying levels of sensitivity to handling procedures, and transcripts can change rapidly in response to stress. Furthermore, RNA degrades at different rates during collection and storage, while a lack of consistent methods for sample preparation, concentration, and quality control complicates cross-study comparisons (Langan et al. 2025; Patin and Goodwin 2023).

The metabolomic analysis of aquatic animals faces multiple challenges that reduce reproducibility. Tissues and matrices exhibit diverse compositions, and biological samples are subject to significant environmental and physiological fluctuations. Moreover, the lack of standardized extraction, analytical, and data-processing systems leads to substantial variations between laboratories and replicates (Salaudeen 2025; Waller et al. 2023).

The iFish database provides multi-*omics* resources which unite genome, transcriptome, epigenome, and proteome data for various fish species but these resources concentrate on genetic and regulatory aspects instead of standardized nutritional phenotype information and diet-based *omics*data (Byrd et al. 2021). The current global nutrition resources for fish primarily present traditional composition information instead of integrated*omics*data (Byrd et al. 2021; Patin and Goodwin 2023). The current global nutrition resources for fish primarily present traditional composition information instead of integrated*omics*data. Critically, while there is strong recognition of the need for community-agreed sampling and reporting frameworks, current efforts remain fragmented. Consequently, standardization in fish nutrition omics remains an aspirational goal rather than an operational reality (Langan et al. 2025; Patin and Goodwin 2023; Salaudeen 2025; Waller et al. 2023).

###  Bioinformatics bottleneck

The genomes of non-model aquaculture species contain multiple genetic segments, such as salmonid whole-genome duplications and molluscan high genetic diversity, which make their DNA structure difficult to understand. Furthermore, limited transcriptomic and proteomic data for these species results in incorrect gene predictions, including splits (fragmented gene models), fusions (incorrectly merged genes), and chimeras (artificial hybrid sequences), as well as insufficient functional information for non-coding and lineage-specific genes (Bachler et al. 2025; Daniels et al. 2023; Hanna et al. 2020; Houston et al. 2020).

Current tools and databases show a preference for model organisms, which results in functional label transfer across extensive evolutionary periods. This often leads to failure when studying fish-specific gene families, including Toll-like receptors (TLRs) and novel immune receptors (Daniels et al. 2023). The “annotation inertia” creates errors which spread between different databases and affect all subsequent analytical steps (Bachler et al. 2025; Nevers et al. 2025). The rapid adoption of high-throughput nutrigenomics and big-data workflows in aquaculture nutrition often outstrips the resources available at local institutions. Reviews from Bangladesh and other aquaculture-intensive, resource-constrained areas show that these regions lack proper infrastructure and bioinformatics training for dedicated staff, and highlight the need for national programs to build an experienced workforce (Rana et al. 2020; Rather et al. 2023). The analysis of fish proteomics becomes difficult because insufficient or poor-quality gene and GO annotation results in three major problems which include the classification of many quantified proteins as uncharacterized and the generation of unstable enrichment results that depend heavily on statistical methods, annotation systems, and the production of weak connections between proteins and biological pathways. The current state of gene and GO annotation prevents scientists from understanding how diet and health and stress responses affect fish biology (Nevers et al. 2025; Yang et al. 2025). To address these limitations, international efforts such as the Functional Annotation of Animal Genomes (FAANG) consortium and its specialized AQUA-FAANG branch are working to improve functional annotation through AI and structure-based tools. However, the advancement rate differs between species and needs both strong reference genomes and specialized bioinformatics expertise to succeed. The current nutrition and proteomics research faces two major challenges because it depends on data that lacks sufficient annotation, leading to incorrect signal interpretation and the omission of essential biological information that applies to fish (Daniels et al. 2023; Houston et al. 2020).

## Conclusion and future perspectives

The development of aquaculture has achieved a major breakthrough through the shift from empirical feeding methods to mechanistic nutrition systems. The review shows how *omics* technologies have started to break down the “Black Box” of fish nutrition which used to measure inputs and outputs without knowing the molecular processes that occurred inside the system. The research evidence presented in this review shows that transcriptomics allows scientists to detect nutritional stress and enteritis markers which appear before growth issues become noticeable through physical changes. The combination of proteomics and metabolomics research shows that gene expression does not automatically result in protein production or metabolic activity which requires scientists to use multiple analytical methods for complete assessment of flesh quality and nutrient utilization.

To unlock the full potential of these technologies, an integrated systems biology approach is required to synthesize their disparate functional outputs into a cohesive analytical framework. The diet-microbiota-host axis which functions as the main controller of fish health requires complete analysis through the combination of metagenomic profiles with host transcriptomic and metabolomic responses. The development of future “Precision Aquafeed” systems needs full comprehension which requires dietary development based on genetic information, life stages, and environmental conditions instead of using conventional nutritional standards. Transitioning high-throughput laboratory technologies to commercial feed mill environments necessitates overcoming several critical bottlenecks that currently hinder their industrial-scale application.

The lack of standardized protocols for sample collection, metadata reporting, and data processing workflows significantly hinders the reproducibility of current research findings. The scientific community requires establishment of universal guidelines which will enable researchers to exchange data between various studies and across multiple species populations. Contemporary *omics* studies in aquaculture primarily demonstrate correlations among parameters, often lacking the mechanistic insights required to substantiate causal relationships. Research in the future needs to develop functional validation techniques which will demonstrate the exact relationships between particular food elements and genetic markers and their effects on metabolic processes. The commercial success of *omics* analysis depends on reducing its current expenses to achieve cost-effectiveness. The development of targeted, benchtop diagnostic tools and specific biomarker panels will be essential for routine industrial monitoring and decision-making. Bioinformatics and AI Integration: Moreover, the increasing amount of data creates an expanding difference between the production of biological data and the process of understanding this data. The process of turning multi-*omics* data into nutritional plans needs two fundamental investments which consist of training bioinformaticians at an advanced level and creating AI predictive models. The aquaculture industry needs *omics* technologies as its essential base which will create a sustainable operation with high efficiency and strong resistance to challenges. The sector will move toward a future which provides exact nutritional needs, active health management, and molecular sustainability assessment through the elimination of current technical and economic barriers.

## Data Availability

No datasets were generated or analysed during the current study.
